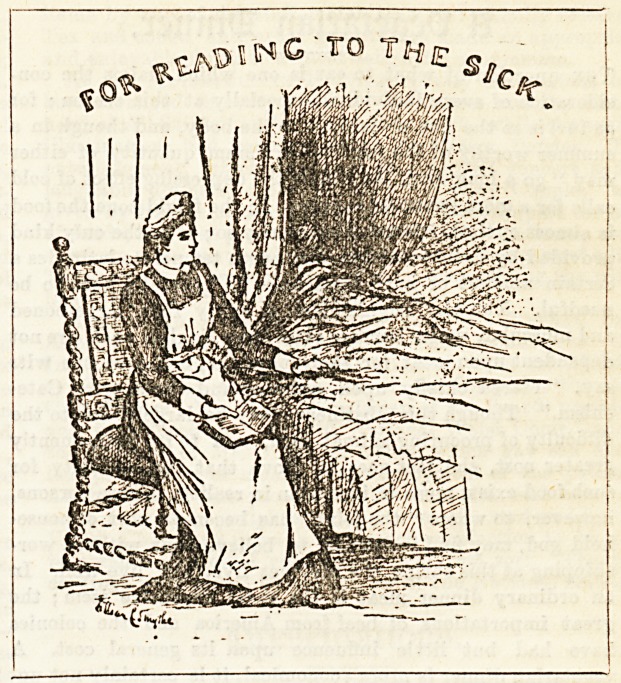# The Hospital Nursing Supplement

**Published:** 1892-01-16

**Authors:** 


### "The Hospital" Nursing Mirror.

Being the Extra Nursing Supplement of "The Hospital" Newspaper.

Contributions for this Supplement should be addressed to the Editor, The Hospital, 140, Strand, London, W.C., and should have the word "Nursing" plainly written in left-hand top corner of the envelope.

### En Passant.

Derby Nursing and Sanitary Association.—We have received the 26th annual report of this Association, which states that its objects are (1) to provide thoroughly educated nurses for the sick—both among the poor and in private families—and (2) to organise means which shall tend to the prevention, and more or less directly to the removal, of disease. The staff numbered at the close of the year sixty-one, including four district nurses and eleven probationers. The death is recorded of Miss F. H. Farnell, for five years a member of the staff. During the year the Queen has become a patroness of the Association, which will henceforth add the word "Royal" to its title.

Presentations.—It is not unnatural that there should be a general outcry against presentations, for they have lately become so numerous as to be a nuisance in the nursing world. In the Army presentations are forbidden; it was found that they were not only an unbearable tax, but that they proved the means of drawing "invidious distinctions," which were not calculated to improve the discipline of a regiment. A correspondent suggests that it is very hard if nurses may not give a small gift to their Matron, and that the fault lies with us who chronicle these gifts, and therefore make them public; but our contention is not so much against the present state of affairs, as against further growth in this direction.

A Christmas Pudding.—If the public only knew it the kitchen of a large hospital on Christmas Day is as well worthy of a visit as the wards are. We wish we could have had a photograph of the big table in the London Hospital kitchen last Christmas morning; on it were 49 puddings, some weighing as much as 20 lbs.; these puddings were to feed 550 persons. The ingredients consisted of 500 eggs, 115 lbs. of raisins, 100 lbs. each of currants and sultanas, 75 lbs. of sugar, 50 lbs. of flour, 40 lbs. of mixed peel, 6 lbs. of almonds, some spice and grated lemon peel, and 12 pints of milk. They were boiled for 16 hours. Hospital housekeeping is a subject we hope to dilate on more this year, meanwhile we merely quote another fact kindly supplied to us by Miss Ransom. On New Year's Day the gift of 40 pheasants, sent by one of the noble patrons of the London Hospital, supplied dinner for 160 patients.

Village Nursing.—We have a good many medical "croaks" from time to time in The Hospital, so we will now have a "crow." Scarlet fever has been entirely stamped out in a Surrey village by the excellent care and nursing of the Reigate Nursing Association. In one case nearly the whole household took the fever, three severely. There was one death in this cottage, but the disease never spread beyond the one house. In another, the eldest boy of a family of four little children—one an infant—took scarlet fever (the fever could be traced in each case from the same infection), and for a particular reason the Hospital for Infectious Diseases could not be used. This boy was isolated, with his nurse, in the cottage, all proper care taken, with the result that not one child took the fever, and he returned to his work, quite well, in due course. Without the nurse all this, in a cottage home, would have been impossible. The Reigate Association has thirteen nurses in constant work, paying each nurse 10s. a week, and giving part of the uniform.

Red-Cross Nurses.—The red cross was the symbol chosen by the Geneva Convention of 1863, by which there was an international arrangement that the wounded in battle should be cared for by a neutral ambulance organization. From this date the red cross has been used by all societies for rendering aid to the sick and wounded in war, hence our own military Sisters are sometimes wrongly called Red Cross Sisters, whereas their proper title is Her Majesty's Nursing Sisters. To make confusion worse confounded, a training school for nurses in Dublin was founded, and has flourished under the name of the "Red Cross Nursing Sisters' House," though it has no connection whatever with nursing in time of war. Save for the misleading name and badge it has adopted, the institution is an excellent one, and has trained as nurses forty ladies. The probationers serve at Meath Hospital and County Dublin Infirmary for a fee of £50, and are afterwards sent out as private or district nurses.

Short Items.—The late Khedive was a firm friend to English nurses, many of whom he decorated with his bronze star. Only last autumn he visited the Alexandria Hospital, and talked to Miss Hamilton and Miss Aird.—The last quarterly letter to the Mary Adelaide nurses is rather a scrappy production. — In his book called "Hospital Children," Mr. Carmichael says: "Practically the nurses are the bulwarks of a hospital."—Miss Florence Nightingale has written to Mr. F. Verney in favour of establishing a School of Health at Buckingham.—The Princess of Wales has sent a cheque for £1,300 to Mrs. Grimwood, the result of the collection made on Mrs. Grimwood's behalf.—Efforts are being made to extend the Leeds District Nursing Association. —Nurse Lees, of the Chertsey Fever Hospital, has been called upon to resign, owing to charges brought against her by a late patient.—The Medical Officer's reports on the insufficient nursing staff of the Exeter Union are still ignored by the Guardians.—The North Oxon Benefit Nursing Association has got into working order.—Miss Gertrude O'Sullivan, Matron of the unlucky Hope Hospital, has been called upon to resign.

Infirmary Nursing.—We are frequently asked if free training in midwifery can be had; we take this opportunity of replying that the Workhouse Infirmary Nursing Association gives free midwifery training to general nurses who bind themselves to serve the Association for two years. The salary they get is usually £30. There are at present two vacancies of this sort waiting to be filled, and candidates should apply to the Secretary, at 6, Adam Street, Strand. Not long since this excellent Association issued a pamphlet, in which it stated: "At present, out of the twenty-four separate infirmaries of London, only half the number employ trained matrons. The position of matron, it is obvious, makes large demands on the administrative faculty, and this can best be met by a supply of women in whom natural refinement is supplemented by superior education and special knowledge. The influence of a lady is felt not only in matters of actual discipline, but also in the maintenance of a high general tone and standard as to work. It is also important that assistant matrons and night superintendents should have received thorough training as nurses." It came on us as an astounding fact that only half the infirmaries had trained matrons; other particulars in the pamphlet with regard to the training of probationers, hours of recreation, &c., were well worth serious study.

### The Expression of the Eye.

It has been said—and by a doctor, too—that the eye itself has no expression, and that the remarks, "His eyes flashed fire," "The depth of the eye," &c., express what is actually impossible.

One does rather wonder, when it comes to be mentioned, how the eye itself can vary in expression, for it is never explained in any of the numerous descriptions of the formation of the eye, its power of distinguishing forms, colours, and so on, with which one so often meets.

Let anyone cover up, as far as it is possible to do so, the surroundings of the various eyes of his acquaintance, and the result will be that they all wear exactly the same expression, or, more properly speaking, they will wear no expression at all.

Or try cutting a hole in a piece of paper, and fitting it upon different drawings of eyes, and the result will be the same. Those who understand drawing will remember that in drawing eyes "the right expression" has always been caught by a little mark of the pencil or brush on the lid or cheek.

Then, what is "the expression of the eye"? for that eyes give a great deal of expression to the face no one denies. Is it that the immediate surroundings give it? The lids, cheeks, forehead, and sides of the nose, whose numerous tiny muscles are pulled in such an immense variety of ways? And if this is so, what is the reason of that bright sparkling of the human eye which we term an "animated expression"? Surely it is the very "animation" that causes it; the eye is thoroughly in action, *animated* in fact, and, therefore, cannot look dull or vacant.

As a matter of course, the "glistening eye" of a person moved either by sad or happy sentiments is caused by the unshed tears which are forced over the ball, and a dry, or "fiery," eye by the unusual absence of any such moisture.

Then, too, how can we account for the assertion that such and such an eye has "no depth"? Some eyes undoubtedly have more depth than others, and this is probably owing to the varying colour. Colour, and shades of colour, vary immensely in different eyes, and it is this variety of shades that gives the so-called thought or feeling of which one so often reads.

"His large dark eyes expressed great depth of thought."

Exactly so! The very fact of his having large dark eyes at all proves that if they expressed anything, and were not wholly vacant, they must express great depth of thought or feeling, love or hate, as the case may be. Who has ever yet met with a large dark eye *in use* that did *not* show great depth of feeling of some sort, be it love or hatred, fun or fear?

Then, again, what a common expression in story books is "The dreamy thoughtfulness of her deep blue eye," "The ineffable depth of love in her dark grey eyes," and other extravagant phrases. Yes; but whoever heard of the "ineffable depth" of love or of anything else in a greeny-white eye?

But if this is so, no one with pale eyes can show any great depth of feeling, and whatever depth of feeling a man or a woman may have, he or she cannot show it unless he has dark eyes!

No; that is—he cannot show it in his eyes. But is it likely that a person with so much feeling should have unfeeling eyes? One would think not; any more than it is likely that a person with a decidedly dark complexion would have yellow or sandy hair.

It is true that there are eyes which vary in colour—looking dark at one time, and nearly white at others—but probably this has a little to do with the light in which they are seen, or it may be that the owners are very changeable themselves. The difference in the size of the pupil, which varies at different times and in different individuals, alters the look, or "the expression," a great deal, and certainly has nothing to do with depth of feeling.

### Everybody's Opinion.

[*Correspondence on all subjects is invited, but we cannot in any way be responsible for the opinions expressed by our correspondents. No communications can be entertained if the name and address of the correspondent is not given, or unless one side of the paper only be written on.*]

### CHRISTMAS FARE IN HOSPITALS.

"L. T." writes: In reading the pleasant notice of the kind treatment shown to the suffering inmates of London hospitals on Christmas Day, the only drawback to my satisfaction was in the case of one hospital (and it may be in others also) to find that whiskey was given in addition to the plentiful feast provided. May I be allowed to protest against such a policy as this, surely as unnecessary as it is dangerous? Even if the doctors can discriminate between those for whom it may be harmless and those for whom it must be injurious, or unsuitable, can it be wise to encourage the taste for stimulants, which all who are working for the benefit of the poorer classes are earnestly endeavouring to check? The taste once revived in the time of weakness may act fatally on many, and it cannot be supposed by any to be an essential element of a Christmas treat. A grand reform has been spread in this respect in many of the workhouses throughout the land, mainly, I may say, through the earnest efforts of women Guardians, and when ladies are numbered amongst the Committees of Management of our hospitals (a "reform" earnestly desired by many), we may look for the like good results there also.

### CONCERTS IN HOSPITALS.

E. M. Davis writes: Much to the delight of the patients, I have had the honour during the year of conducting 34 concerts at the Royal Free Hospital. Among those who have so kindly and so ably taken part are the following: Madame Florence Winn, Miss Hannah Jones, Madame Marion Burke, Miss Adela Bona, Miss Lizzie Jones, Miss Gwen Davies, Miss Fort, Miss Chapman, Miss Maggie Pritchard, Miss Snell, Miss Katie Williams, Mrs. Tweedie, Miss Hepburn, Mrs. Beachcroft, Miss Elsie Mackenzie, Miss Strathearn, Miss Rose Kirkham, Miss Myganwy Williams, Miss Sharer, Miss Hammond, Mr. Dan Price, Mr. Curtis, Mr. Cousins, Mr. Bamberger, Mr. Harris, Mr. Ellerbeck, Mr. Covington, Mr. Odell, Mr. Merrylees, Mr. Math. Jones, Mr. Salmon, Mr. Tudor Rhys, Mr. Lloyd Williams, Mr. Chapman, Mr. Arnold, Mr. Hammond, Mr. Blennerhasset, Mr. Short, Mr. Watson. This is work that angels may well covet; and work, too, that affords pleasure of the highest type. The late Madame Annie Williams, having sacrificed a lucrative professional engagement in favour of the patients of the London Hospital, on her return home remarked: "In singing to the patients this evening I experienced the greatest joy of my life." "Inasmuch as ye did it, come, inherit the kingdom."

### QUEEN'S NURSES.

"I. W." writes: In your issue of last week I observe an advertisement for probationers with previous training from the Queen Victoria Jubilee Institute. As one interested in the success of so great a movement as that of the Institute, may I venture to express some surprise that the advertisement in question requires written applications to be sent to the Secretary, who is, I understand, a gentleman? This appears a curiously retrograde system, calling to mind the advertisements of some Poor Law Infirmaries in which nurses are still desired to apply to the Medical Superintendent. It seems a pity that the Jubilee Institute should not follow the example of the best nursing institutions, and arrange that applications should go direct to the person best able to deal with them, namely, to the trained lady who is from experience most fitted to consider them on their merits, and who can easily judge of the previous training of candidates from a wide knowledge of the various training schools. I write without knowledge of the reasons for the wording of this advertisement, and with the simple desire that the Jubilee Institute should command a large number of first-rate applications.

### Real Scandals.

MANY are the attacks made on our voluntary hospitals, and presumably with the desire of placing all these institutions under Government control, and making them rate-supported. Yet the real scandals in our experience generally occur in rate-supported hospitals, particularly in those for fever cases, to which, of course, the public cannot have free admission. Pages of printed complaints are devoted to the fact that the ordinary hospital nurse has to work twelve hours; but the fact that the nurse of the Brentford Union stated to the Guardians on January 6th, that she was so overworked she had not been to bed since December 20th, is passed over as a matter of no account. We would recall the fact that last January, when the appointment at Brentford was vacant, a trained nurse who had applied for the post refused it when offered, because she found she was expected to attend to 150 patients, which, she said, she "could not conscientiously undertake to do"; therefore another applicant, whose suitability consisted in her statement that she "had once read with a view to taking medical examinations" was appointed.

The grossest neglect of patients and nurses alike seems to occur in many workhouses; would that the Workhouse Infirmary Association had more money, and matters would soon be remedied. Dr. Woodman, on January 5th, reported to the Exeter Guardians on the continuance of the difficulty of getting efficient pauper ward attendance, especially on the female side. When wardsmen or women, said the Doctor, had worked all day it was impossible for them to be ready to jump out of bed at all times of the night aiding the bed-ridden sick. A night nurse was therefore required to visit the wards in turn and attend to the wants of the patients. He would also call the attention of the Guardians to the number of sick males—twenty-six—still without supervision in the annex ward; he advised once more the building of a wing on the male side of the hospital. Mr. Munro remarked that "this doctor's report" had become almost a monthly affair; and the Committee had been so thoroughly into the matter on former occasions that in the present instance they thought it undesirable to make any recommendation. Mr. Waller thought the doctor should be reproved. It was ridiculous of him to occupy needlessly so much time. Mr. Andrew asked for the report of the doctor to be re-read. This being done, he remarked that he did not think it right that the Board should incur the onus of treating with silence such a serious and important report. Mr. Munro said there was no doubt the inmates were excellently cared for. The doctor no doubt made these reports so continuously so that in case any accident should happen the brunt of it would be on the Guardians. Well, he thought the Guardians' back was strong enough to bear the strain.

It will not be long before there is a scandal at Exeter.

### Appointments.

SOUTHEND LOCAL BOARD HOSPITAL.—Miss S. W. Doughty has been appointed Matron of this hospital. She trained at the Norfolk and Norwich Hospital, and stayed there five years; for one year she has been Sister of the male wards at Chichester. Miss Doughty holds excellent testimonials.

PAISLEY.—Miss M. A. Hempseed has been appointed District Nurse at Paisley. She trained at Greenock Infirmary, and has worked for three years for the Glasgow Sick Poor and Private Nursing Association, where she has won golden opinions.

INDIAN SERVICE.—Miss Grace Wood has been appointed a Sister in the Indian Nursing Service, and sails on January 28th. Miss Wood trained at Cardiff for three years, and has worked at Newport and elsewhere. We wish her success in her new sphere.

EDINBURGH ROYAL MATERNITY AND SIMPSON MEMORIAL HOSPITAL.—The directors, at their meeting on the 11th inst., appointed Miss Catherine Edward Matron of this hospital vice Mrs. Mather resigned.

### ANNIVERSARIES.

WE have been passing through a season in which there were many anniversaries, and perhaps we have felt unhappy in consequence, for it seems so hard that we should be lying on a sick bed while those around us are enjoying a bright and merry time. There are a good many ways in which we may look at even this dreary picture. Probably we have had our turn of prosperity and were very happy last year, going on carelessly and never imagining that in twelve months' time we should be laid low. I wonder whether this happiness softened our hearts and made us sympathising and considerate then for those who were in sal estate? If not, our present afflictions are no doubt sent to teach us those Christian graces.

We are all more or less selfish and require that God should take away our daily mercies for a time in order that we may throw self on one side and learn to mourn, with those that mourn, when we are light hearted.

We may make our anniversarsies now and always a cause of pleasure or profit. Let us look at those we have been celebrating. First the birthday of Christ. Was He not born to save us from our sins? Here then is a matter for endless rejoicing, while our own birthday should fill us with thankfulness to God who made us reasonable beings, put us on a beautiful earth in which to spend our lives, and gave us the promise of a blessed eternity after death.

Then a New Year is an anniversary which gives us an opportunity to make new resolutions which should not be let slip. It is far easier to give up a bad habit when there is some reason, such as the beginning of a twelvemonth, to make a fresh start; or we may resolve to be more patient, to be more cheerful, to give a smile for a frown, to cultivate the soft answer which turneth away wrath. These good resolutions we must have help to keep, and our dear Lord will give it us if we ask Him for it.

There are some days which can only bring sad memories to our minds, such as the death of friends; these griefs time alone will assuage; but if we look on the bright side of things even some misfortunes have compensations. For instance, we recall that this day two years we broke a leg, but what a life-long friend we gained in the surgeon who set it so skilfully; and to come nearer the present, we were stricken down with a fever this day two months, but shall always remember with gratitude the day we were brought into the hospital, where we have received such tender care that it is predicted we shall have better health in the future than ever before in our lives. So love to God and man will gild the hour which had, at first, appeared so black and dreary.

### A Vegetarian Dinner.

THE question of what to eat is one which claims the consideration of every individual, especially at this season; for as fuel is to the fire so is food to the body, and though in a summer worthy of the name a minimum quantity of either may "go a long way," in winter the depressing effect of cold calls for a more generous supply. In the frigid zone the food is almost entirely animal, necessarily so; it is the only kind provided by nature; and in our north temperate latitudes a certain amount of such sustenance is generally held to be needful. Yet the Highlanders, a hardy race, large-boned and muscular, under harsher skies than English ones, are not dependent upon meat for their physique, being, so the wits say, "reared chiefly upon oatmeal and the shorter Catechism." Though this abstinence may be largely due to the difficulty of procuring animal food, and to its consequently greater cost, the fact goes to prove that the necessity for such food exists more in idea than in reality. Those persons, however, to whom "the joint" has become a sort of household god, may find it difficult to believe that without worshipping at this shrine one may yet live, and live well. In an ordinary dinner meat is the most expensive item; the great importations of beef from America and the colonies have had but little influence upon its general cost. A vegetarian dinner is more economical, it is certainly not unwholesome; and provided one can add the due proportion of that "best sauce" which is necessary to the enjoyment of all food, to wit, "hunger," it is quite as agreeable and as satisfying as need be. Any one may prove the truth of this observation by dining, as we recently did, at the "Acmé" Vegetarian Restaurant in Gray's Inn Road, where a good hot dinner, nicely served, and excellent both as to quantity and quality, costs sixpence. For this modest sum we had, like a certain right hon. gentleman, three courses open to us (unlike him, we took them all), soup, savoury, and sweet, besides an unlimited allowance of white and brown bread. The *menu* included ground rice and milk, brown onion and lentil soups, potato and onion pie, pease pudding, tomatoes and haricots, green vegetables of all kinds. The name of one dish, "Midlothian steak," piqued our curiosity, and we felt in duty bound to try it. It consisted of a slice of pudding in which oatmeal was conspicuous, highly seasoned and served with rich brown sauce and vegetables. There were sweets galore, such as milk puddings and jam, and stewed fruits, figs, damsons, prunes and apples. The guests all belonged to a respectable class. They could not be called fashionable, silk hats were in ignominious minority. There was a marked absence of men of the Cassius type with that "lean and hungry look," which is apt to be associated with the devotees of this cult in the minds of the general public. Why this fallacy still exists is not very clear. An old and well-known book gives an account of an experiment once made under the most favourable conditions, to prove the merits of the rival systems of vegetarian and omnivorist; and it is stated that the verdict was in favour of the former, "their countenances appeared fairer and fatter in flesh than all the children which did eat the king's meat."

Now a dinner equal to that given in the Acmé, in quantity and variety, but of which meat formed a part, would cost, if not double the money, at least one-half more, and few of us can afford to ignore the question of expense. Working people, as a rule, have but slender purses to consult, and it is, therefore, of moment that they obtain the greatest possible amount of good with the means at their disposal, consistent with a proper maintenance of bodily strength to fit them for duty. Vegetarian restaurants are, we presume, to be found in all parts of London and other great centres. To those of our readers who have not yet overcome their prejudices sufficiently to patronise one, we recommend a trial visit.

### Keeping Christmas.

ST. MARY ABBOTT'S INFIRMARY, KENSINGTON.—On Christmas Day the Matron (Miss Hughes), and the Assistant Matron (Miss Griffiths), who has been appointed to the charge of the temporary establishment at Plaistow, were the gratified recipients of two handsome testimonials subscribed to by the nurses of the Infirmary, who assembled in full strength in the principal mess-room to show the great and kindly esteem felt for their superiors. The gift to the Matron, presented by Staff-Nurse Poulter, consisted of an exceedingly handsome set of silver knives and forks, and that to the Assistant Matron, presented by Staff-Nurse Simmons, of a very pretty set of silver spoons, sugar tongs, &c. Both presents were suitably acknowledged, and, being entirely unexpected, were all the more appreciated. On the Monday and Tuesday Christmas trees, profusely bedecked with appropriate gifts, were provided for the smaller inmates by the kind generosity of Miss Wells, of Midhurst. Among those who assisted in the distribution of the numerous presents, and helped to supply pleasant entertainment for the children, were the Misses Linnell, the Misses Lowndes, Mr. H. and Mrs. Potter, Mr. Chilcott (assistant medical officer), Mr. C. W. and Miss Cunningham, Miss Hughes, Miss Griffiths, &c.

HER MAJESTY'S HOSPITAL, STEPNEY, was prettily decorated for Christmas, mottoes, wreaths, flags, &c., having been arranged in the wards and corridors, giving to all a very cheerful appearance. The patients, sixty-two in number, awoke at an unusually early hour to find on the foot of each bed useful articles or toys, to suit their various ages, which ranged from a few weeks to twenty-two years. The toys were provided from the *Truth* collection. The dinner of roast beef and plum pudding, followed by oranges and apples, was heartily appreciated by those who were able to partake of it, and later on in the afternoon a bran pie, containing all kinds of small comical articles, was carried round by two of the nurses, who dressed up as Father Christmas and an old woman. On the 5th inst. a tree was held in the Gordon Memorial ward on the surgical landing, through the kindness of Mrs. B. A. Miller, who, with several friends, spent the day in dressing the tree. She gave each patient one or two nice presents and a new piece of money. She also provided cakes and jam for tea, after which a very good conjuror and ventriloquist caused great amusement. The tree was kindly presented by Mr. T. Fowell Buxton. This hospital, which contains seventy-five beds, is in connexion with Dr. Barnardo's Homes. The Matron, Miss S. A. Warburton, was presented on Christmas Day, by her nurses, with a very handsome tea set, which she values the more highly as a further mark of the good feeling existing in the hospital.

On the last night of the old year an entertainment was given by the staff of the Dundee Medical School to the patients in the DUNDEE ROYAL INFIRMARY. The spacious and well-appointed accident ward was transformed to suit the occasion, a commodious stage having been erected at one end, while the opposite end was occupied with a twelve feet lantern screen. Dr. McCosh, the Medical Superintendent, presided. Every person in the institution who could be present, and every patient who could walk or bear to be carried, were congregated to enjoy the medley of mirth and melody which had been provided. The programme opened with pianoforte selections by Nurse Shaw, which were much enjoyed. The vocal selections included several songs by Miss Resch Petterson, all of which were rendered with exquisite taste. Nurse Garvie's singing of "Love's Old Sweet Song" was also well received, as was also Nurse Rough's song "There was a Lad." The students were particularly successful in their negro impersonations. The songs and choruses were well selected and effectively rendered, while the impromptu hits and extravaganzas were received with much enthusiasm. The fact that their stage had a foundation of ward beds served only to enhance the musical effect. Miss Murison acted as accompanist during the evening with much acceptance. The lime-light lantern proved a pleasing variation, a large number of slides being shown by the dispenser.

THE COTTAGE HOSPITAL, BLANDFORD.—The first Christmas spent in this new and beautiful little hospital proved a very] bright one. The wards were prettily decorated. Christmas cards greeted the patients' eyes in the early morning. A dinner of turkey, plum pudding, and mince pies, provided by the Hon. Miss Portman, was thoroughly enjoyed. The tea was a right merry meal, ten out of the eleven patients being able to assemble together with some of the children of patients in the hospital, and the entire staff—Matron, two nurses, servant, and porter. Then followed a Christmas tree, the gift of Viscountess Portman. After singing some Christmas hymns the day closed, and by general consent was voted one of the happiest ever spent. On Tuesday, December 29th, a magic lantern entertainment was given to patients and some who had been inmates during the year, making a total of forty. The Christmas tree again afforded pleasure, most of the guests being children.

On Tuesday evening (December 29th) the annual treat to the patients and ex-patients took place at CONGLETON HOSPITAL, under the management of Miss Wagnell, the respected Matron, and was in no way less enjoyable than in previous years. After the tea was over, several ladies and gentlemen entertained the patients with music, singing, &c., then "Santa Claus" (Mr. C. R. Hall) made his appearance with bags of gifts. Before the distribution took place he was deputed to make a presentation to the Matron.

AT GLASGOW ROYAL INFIRMARY on New Year's Day there was the usual pleasant gathering of nurses and their friends. The speakers included Sheriff Guthrie and Sir James King, but the Lord Provost made the most stirring speech, and in obvious reference to the late attack on the Infirmary said:—"We may frequently fail to give satisfaction to those around, and may often be constrained to acknowledge to ourselves that we have fallen far short of what we wished to accomplish. We may be judged unkindly, unjustly, and ungenerously by those who are themselves doing little or nothing for the good of their fellow-men; but if we have done our very best to perform conscientiously and to the utmost of our ability all the work entrusted to us, we may with confidence hope that the verdict of the great Master who knows the secrets of all hearts will be, 'Let her alone; why trouble ye her? She hath wrought a good work on me; she hath done what she could.' (Applause.) I trust each nurse in looking back on the work of the past year may have a clear conscience in regard to the treatment of her patients, and may be able to say, 'The Master knows I have done what I could.' I hope this may be true of all our nurses, and in that case this will be to them a very happy New Year's Day indeed."

At the JENNY LIND INFIRMARY there was a Christmas tree, a delightful Father Christmas, and a dear little clown to distribute the presents. There were gifts for every child in the hospital, and the decorations were successful, thanks to the efforts of the Matron and nurses.

Owing to the kindness of Lady Macdonald Lockhart and family, Christmas and New Year's Day are always festivals at the LOCKHART HOSPITAL, LANARK, but this year there were extra rejoicings, as the Matron had completed her 20th year of service amongst them. On Christmas Day a large party of children, old patients, and present patients' friends were invited to tea, and after some hours of play and fun, some of the local clergy assisting and some of the ladies from the Lee, they all got something useful from the large tree, and a bag of good things away with them. The Matron got a great many presents from friends of the hospital and Lady Lockhart, also from the servants and patients, which was very gratifying to her.

THE COTTAGE HOSPITAL, ST. HELEN'S.—On Christmas Day the patients had a tea party, each one inviting a friend—about 50 in all. Tables were laid down in one of the larger wards and spread with all sorts of good things. At seven the concert began, given entirely by the staff. Between them they made up a programme consisting of songs, recitations and music for piano, violin, and banjo. They finished up at nine with "Auld Lang Syne" (all joining hands), and a distribution of gifts. Each patient had a parcel of useful things, and each visitor some trifle as a souvenir of the hospital. Last Tuesday, the 5th of January, was, however, the grand field day, the day of the annual concert. The accident ward was cleared and draped, and looked charming. They prepared for a hundred people, but owing to the horrid weather there were not more than eighty in number. The programme consisted of songs, vocal duets, and quartettes, violin solos and duets, piano and banjo solos. The music was provided by friends from outside, with the exception of some items by one of the staff, which were very kindly received. Tea and coffee, and other good things made an appropriate and enjoyable finish to a most delightful programme.

At the METROPOLITAN HOSPITAL pretty decorations had been arranged in every ward by the nurses and the Sisterhood of All Saints', under whom they work, while the varied illuminations had been presented by Messrs. Defries. The entertainment, which consisted of a capital concert and a laughable dramatic piece, entitled "A Cosy Couple," was given by friends of the charity in a ward unoccupied for lack of funds, and was greatly appreciated by the patients, some of whom were conveyed to it, beds and all, by means of a lift. Those whose ailments precluded them from joining the party had their loss made up to them in other ways.

A MUSICAL EVENING AT THE LONDON CENTRAL SICK ASYLUM, CLEVELAND STREET, W.—The inmates of this Institution had a capital treat afforded them on Monday evening last at the instigation of the Chaplain, the Rev. C. P. Baxter .Mr. Hobbs (Chairman of the Board) presided, and introduced the "Cardiff National Welsh Choir," under the direction of Mr. C. Em'yn Jones, which gav ean excellent programme of music, to the enjoyment of the patients. At the close of the proceedings the chaplain said everyone must have been delighted with the music rendered by the Welsh Choir, and it was a matter of satisfaction to be able to arrange an annual entertainment of this sort. The proceedings were closed by the singing of the National Anthem.

### Presentations.

ON New Year's Day, at the Whittington Hospital, a handsome tray and china tea service was presented to Miss Sibley, the Lady Superintendent, by the nurses and probationers, as a mark of their appreciation of Miss Sibley's kindness, courtesy, and interest in their welfare. The presentation was made by Dr. J. S. Orchard, who spoke of the immense improvement in the Hospital generally since Miss Sibley's appointment to the post of Lady Superintendent.

MR. J. P. RICHARDS, on leaving Hanwell Asylum, was presented by the nurses with a handsome dressing bag.

OLDHAM INFIRMARY.—The nurses presented Miss Simpson, the Matron, on Christmas morning, with a pair of silver salt-cellars and silver sugar-tongs.

ST. HELENA HOME.—On Christmas Day the nurses of St. Helena Home presented their Lady Superintendent, Miss Robertson, with a silver Queen Anne sugar-basin, cream jug, and tongs, in a case, as a mark of their sincere regard and appreciation of her continued kindness to them.

### Death in Our Ranks.

NURSING SISTER M. GARDENER, Army Nursing Service, died January 2nd, 1892, at the Royal Military Infirmary, Dublin. She had nearly completed five years' service. She was accorded a military funeral.

### Notes and Queries.

### Queries.

*Publisher Wanted* of "Florence Nightingale, the Wounded Soldier's Friend," by E. F. Pollard.—*S. J.*

*Screens.*—Where can I get bamboo frames for folding screens?—*K.B.M.*

### Answers.

*E. D. J.*—Of the two we should say Melbourne offered the best likelihood of work, but we can only refer you to our Australian correspondence of the last three years.

*Lydia.*—No; you will have to pay, unless you join the Workhouse Infirmary Nursing Association, 6, Adam Street, Strand. See our advertisements.

*E. A.*—You will have to begin again if you wish to gain a London certificate. We advise you to answer our advertisements, or to apply to any large hospital of over 100 beds.

*M. D.*—We shall be glad to use your lines, "To a Nurse," when we have room.

*Dispensing.*—Get "Practical Dispensing," by C. J. S. Thompson; or it is wise to ask at the school what handbook they advise.

*Christmas Parcels.*—Through a mistake, two pairs of socks from Sister R. Turner were not acknowledged before.

*Christmas Competitions.*—We have sent £1 to Nurse Ayrton, 41, Russell Road, Kensington; 10s. to Miss McEwen, The Gables, Southport; 5s. to Miss Mable White, Leighton, Southsea; 5s. to Nurse Firth, Ivy Cottage, Wakefield; 10s. to Miss Kemp, The Briars, Sandown; and 10s. to the Manager of the Royal National Pension Fund for the Benevolent Branch; being the prizes won in the Christmas Competition. Miss Hale requested that her prize might be sent to the Pension Fund.

### Told on New Year's Eve.

(*Concluded from page* lxxxiv.)

Mechanically, I followed Sister, feeling as if it were a dream, and feeling no surprise. It all seemed as if I had been waiting *just* for this, until we reached the ward, where on a bed in a screened-off corner lay a man, worn with illness, and with *that* look in his face we knew only too well, and yet, wasted and worn as he was, I knew him, only I could not speak—could only stand motionless waiting for what was coming.

"Muriel," he gasped, and feebly held out his hand; and yet I could not take it. I could only wait. The night nurse moved away, and we were alone; and the bells still rang.

"Muriel," again, he said, "I have come. I hardly thought to have reached here alive. I knew you were here. It is no chance. I——" Here a death-like faintness came over him, but reviving after some stimulant, went on—

"I want to tell you. I want to ask you to forgive me. You remember, it was five years ago."

Remember!—and there rose before me a vision of a snow-clad ground, the grand old porch of a country house, and a girl standing, watching the retreating figure of a horseman going down the avenue, and then turning to go in, a glad light in her eyes, as she whispered to herself "to-morrow"—that morrow that only came in sadness.

A sign of assent was all I gave. How strange everything seemed. Should I awake suddenly and find myself on my little bed in the dormitories? But no; it was all real, and Jack Leslie—he was a reality, worn and ill as he was.

"Muriel," he went on, "you remember that day—the day Guy left, and you expected him the next day—he said he was going—well, but I must make it short, I cannot speak long. You know who were at Clere Hall, and though Guy came over early I did not see him to speak to till after dinner in the smoking-room. I saw he had been vexed; he was very silent until—until we were alone, he suddenly said, 'Tell me it is not true,' and on my asking what he meant, went on 'That you are engaged to Muriel Yorke'—and oh, Muriel, I never forget how he looked at me."

"And you! What did *you* say?" I cried, finding my voice for the first time—and how far away it seemed.

"I said nothing" was the answer; "but did as bad. I let him think I was."

"Did—did *she* tell him?"

"Mine alone be the blame," answered Jack, "she suffered as I have. And Guy—where is he now? I heard he had gone abroad."

"Why—why—did you?" I cried. "And you were always his friend and mine, it was cruel—cruel—"

"Because," he interrupted me, "I was weak, and let myself be persuaded, and because—Oh, Muriel—did you never guess it?—I cared for you—I thought more than Guy did, but it was not true, for he loved you madly, and you know how the Christmas party had suddenly been broken up, and we all were scattered,—do not cry," he went on, as the bitter tears coursed down my cheeks as I thought of all the deceit that had been practised, and what Guy must have thought of me. "God knows I have repented, and then this illness came on. I knew you were here, and I felt I could not die until you had forgiven me"—and here we were interrupted by the night Sister. She wanted to know if Jack would like to see a clergyman, but he said "No," and we were again alone.

"Muriel, Muriel," he cried, in a voice already fainter, "say you forgive me for having spoiled your life. I had hoped until quite lately that you and Guy had met, and that you were happy. Forgive me for not saying it was a lie—and he stretched out his hand, already damp with the death dews, and gazed with longing, troubled eyes in my face.

But I was still silent, it all seemed so horrible—that deliberately planned-out deceit—and then the years that had passed—and yet—my life had not been spoiled—and Guy—he had only this year married in Australia—and Jack—Jack who had been once a sort of brother—Jack was dying—

"Forgiveness to the injured doth belong." I knew that, and—

"Muriel, tell me"—and there seemed for a few seconds a deathlike silence, and then clearly and distinctly sounded the great clock of St. Paul's, telling that year had gone, and a merry peal then ushered in the new one; and with them a great feeling of pity rose in my heart for the man lying before me—everything seemed left behind; there was only the present now—only Jack, whose one cry before he left this world was for my forgiveness—and we had been such friends once. I could only think of him as then, and so kneeling down beside him said—

"I do forgive you, Jack," and as a look of unutterable peace stole over the dying man's face, went on, "And you have not spoiled my life, everything is as it should be."

"And Guy will know," he murmured.

"No, he will not," I answered, "he will not want to—time heals all wounds, and he is married and happy."

"Muriel, say it again; say 'I forgive you,' and say you are not unhappy."

"I forgive you Jack, and I am content now"; and bending down kissed him.

And so we remained, talking a little at intervals—until the Great Messenger came, and Jack Leslie, with a smile on his lips, passed beyond.

It has ended now, and I knew the reason why, and when, the last sad offices for the dead performed (I would let no one else do them but myself), and I was again in my room and could think over it all, the only feeling of bitterness that arose (and it was but a momentary one) was against—no, I will not say whom; he said she had suffered, and Guy had married, and I—I was content with my life. Early left desolate without many relations, I had found a home and work, a work that brought its own happiness. It was thinking of Jack that the sadness consisted in—thinking of his wasted life. Well! perhaps not that since "Heaven is made for beaten men."

It was with a feeling of relief that I was told at six a.m. that I need not go on duty, and so I lay most of that New Year's Day thinking over many things. To say there was not some "ache" over it all would have been "one big lie," but still it was not all that.

"You *are* late, Nurse," said my patients, as I entered the ward in the evening in the capacity of guest. "Fancy wishing us a 'Happy New Year' now; why it is quite old by this."

And again a few years have passed, and I am Sister St. Monica now—still content in my life—a desolate woman "set in a family"; and I have seen Guy again, and we met like old friends, making no allusion to the past (only I *knew* that he had learned the truth), and somehow the only grief that remained was in the memory of "Jack Leslie."

## Figures and Tables

**Figure f1:**